# The outcomes of thoracoscopic decortication between fungal empyema and bacterial empyema

**DOI:** 10.1186/s12879-022-07978-z

**Published:** 2023-01-06

**Authors:** Ya-Fu Cheng, Chun-Min Chen, Yi-Ling Chen, Ching-Yuan Cheng, Chang-Lun Huang, Wei-Heng Hung, Bing-Yen Wang

**Affiliations:** 1 Division of Thoracic Surgery, Department of Surgery, Changhua Christian Hospital, Changhua, No. 135 Nanxiao St., 500 Changhua City, Taiwan; 2 Big Data Center, Epidemiology and Biostatistics Center, Changhua Christian Hospital, Changhua City, Taiwan; 3 Surgery Clinical Research Center, Changhua Christian Hospital, Changhua City, Taiwan; 4 Department of Post-Baccalaureate Medicine, College of Medicine, National Chung Hsing University, Taichung, Taiwan; 5 School of Medicine, Chung Shan Medical University, Taichung, Taiwan; 6 School of Medicine, College of Medicine, Kaohsiung Medical University, Kaohsiung, Taiwan; 7 Institute of Genomics and Bioinformatics, National Chung Hsing University, Taichung, Taiwan; 8 Center for General Education, Ming Dao University, Changhua City, Taiwan

**Keywords:** Bacteria, Empyema thoracis, Fungus, Outcomes, Surgery

## Abstract

**Background:**

Fungal empyema is an uncommon disease and is associated with a high mortality rate. Surgical intervention is suggested in stage II and III empyema. However, there were no studies that reported the outcomes of surgery for fungal empyema.

**Methods:**

This study is a retrospective analysis in a single institute. Patients with empyema thoracis who underwent thoracoscopic decortication between January 2012 and December 2021 were included in the study. We separated the patients into a fungal empyema group and a bacterial empyema group according to culture results. We used 1:3 propensity score matching to reduce selection bias.

**Results:**

There were 1197 empyema patients who received surgery. Of these, 575 patients showed positive culture results and were enrolled. Twenty-eight patients were allocated to the fungal empyema group, and the other 547 patients were placed in the bacterial empyema group. Fungal empyema showed significantly longer intensive care unit stay (16 days vs. 3 days, p = 0.002), longer median ventilator usage duration (20.5 days vs. 3 days, p = 0.002), longer hospital stay duration (40 days vs. 17.5 days, p < 0.001) and a higher 30-day mortality rate (21.4% vs. 5.9%, p < 0.001). Fungal empyema revealed significantly poorer 1-year survival rate than bacterial empyema before matching (p < 0.001) but without significant difference after matching.

**Conclusions:**

The fungal empyema patients had much worse surgical outcomes than the bacterial empyema patients. Advanced age and high Charlson Comorbidity Index score are independent predictors for poor prognosis. Prompt surgical intervention combined with the use of antifungal agents was the treatment choice for fungal empyema.

**Supplementary Information:**

The online version contains supplementary material available at 10.1186/s12879-022-07978-z.

## Background

Empyema is a serious infection with increasing incidence. Previous studies reported that the 30-day mortality rate of empyema thoracis was about 6.5 to 10.6% after surgery. [1–3] Fungal empyema is an uncommon disease associated with high crude mortality. The most common pathogens of fungal empyema are from the *Candida* species. Candidemia was diagnosed in only 2 to 27% of patients with empyema in a previous report, but the mortality rate of fungal empyema was reported to be over 70% [4, 5].

To our knowledge, there have been only 3 studies that reported the outcomes of fungal empyema [6–8]. However, there was no report on the outcomes of fungal empyema after surgery. Consequently, there was no comparison of surgical outcomes between fungal empyema and bacterial empyema either. The treatment for fungal empyema thoracis remains controversial and is not protocolized.

There are three stages in the natural course of empyema: the exudative stage (stage I), fibrinopurulent stage (stage II), and organizing stage (stage III) [9]. Surgical intervention is suggested in stages II and III to remove fibrin and peels. The main goals of surgical therapy are to control infection by evacuating pus from the pleural cavity and to improve lung expansion by removing pleural peels from a trapped lung.

In this study, patients with empyema who received thoracoscopic decortication of pleura between January 2012 and December 2021 in our institute were enrolled. We compared fungal and bacterial empyema in terms of patient characteristics, risk factors and outcomes. Our goal was to find the risk factors and prognostic factors of fungal empyema. We hoped to improve the treatment strategy and provide better surgical outcomes.

## Methods

### Patient population and selection

This study is a retrospective analysis in our institute (Changhua Christian Hospital, Changhua, Taiwan). All patients with empyema thoracis who were over 18 years of age and underwent thoracoscopic decortication between January 2012 and December 2021 were included in the study. Patients with missing therapy records, recurrent empyema, or negative results for both the pleural peels culture and pleural effusion culture were excluded.

Our study was approved by the institutional review board in our institution (IRB-220601), and informed consent from all participants was waived. We analyzed the age, gender, smoking status, Charlson Comorbidity Index (CCI) score, phase, pathogen, location, cause of empyema, and laboratory data. The phase of empyema was identified by two doctors (YF Cheng and BY Wang) via a chest computed tomography image and an intra-operative photo and blinded to the outcome. The cause of empyema was reviewed from the chart record and it was decided by the attending doctors of those patients. The iatrogenic empyema was defined within 30-day after previous surgery. All of the cultures were done during the operation. We separated the patients into a fungal empyema group and a bacterial empyema group according to the culture results. If the pathogen included both fungus and bacteria, the patient was assigned to the fungal empyema group. For bilateral empyema, we only did the culture at more severe site. We suggested that patients with bilateral empyema were originated from the same pathogens. We used 1:3 propensity score matching to reduce the selection bias in this observational study. Age, gender, smoking status, CCI score, phase, pathogen, location and laboratory data were used for the propensity score matching. The primary outcome measure for our study was 1-year survival rate. The secondary outcome measures included 30-day mortality and the durations of intensive care unit (ICU) stay, hospital stay, and ventilator usage. The overall survival (OS) was calculated as the time from operation date to either death as a result of any cause or the April 2022 cutoff. It’s because of more than half of the mortality event happened within 4 months after surgery. A patient was transferred to ward from ICU after weaning of ventilator and stabilization of vital signs.

### Statistical analyses

Survival curves were plotted by the Kaplan–Meier method. Univariate and multivariate analyses were performed with the Cox proportional hazards model. The following factors were included into analyses: pathogen, age, gender, smoking status, CCI score, phase and location. We used the chi-squared test to compare differences between categorical variables and the t test to compare continuous variables. All calculations were performed using IBM SPSS Statistics for Windows, Version 22.0 (IBM Corp., Armonk, NY). Statistical analysis with a p value less than 0.05 was considered statistically significant.

## Results

In this study, 1197 patients with empyema received thoracoscopic decortication. Of these, 575 patients showed positive culture results in a pleural peels tissue culture or a pleural effusion culture and were included for evaluation. The culture positive rate was 48%. Twenty-eight patients were allocated to the fungal empyema group, and the other 547 patients were placed in the bacterial empyema group.

The clinicopathological characteristics of all study patients before matching are shown in Table 1. The mean age was similar in each group (65.50 years old in the fungus group vs. 62.64 years old in the bacteria group, p = 0.358). Both groups were male predominant (92.9% in the fungus group vs. 77.9% in the bacteria group, p = 0.059). The proportion of ever smokers was 46.4% in the fungus group and 28.5% in the bacteria group (p = 0.042). Most of the patients had many comorbidities and high CCI scores (CCI >  = 3: 60.7% in the fungus group vs. 51.2% in the bacteria group). The detailed data of CCI scores in both groups were showed in the Additional file 1: Table S1.

**Table 1:** Basic characteristics of the empyema patients

	Fungal empyema	Bacterial empyema	*P*
Number of patients	28	547	
Age (years) mean ± SD	65.50 ± 11.91	62.64 ± 16.20	0.358
Gender			0.059
Male	26 (92.9%)	426 (77.9%)	
Female	2 (7.1%)	121 (22.1%)	
Smoking			0.042
Never	15 (53.6%)	391 (71.5%)	
Ever	13 (46.4%)	156 (28.5%)	
CCI score			0.528
0	3 (10.7%)	97 (17.7%)	
1 ~ 2	8 (28.6%)	170 (31.1%)	
> = 3	17 (60.7%)	280 (51.2%)	
Phase			0.390
II	23 (82.1%)	410 (75.0%)	
III	5 (17.9%)	137 (25.0%)	
Location			0.004
Right	20 (71.4%)	331 (60.5%)	
Left	6 (21.5%)	211 (38.6%)	
Bilateral	2 (7.1%)	5 (0.9%)	
Pathogen			0.001
Single	6 (21.4%)	398 (72.8%)	
Multiple	22 (78.6%)	149 (27.2%)	
Cause			< 0.001
Pneumonia	13 (46.4%)	443 (81.0%)	
From abdomen	1 (3.6%)	16 (2.9%)	
From neck/mediastinum	1 (3.6%)	1 (0.2%)	
Cancer related	2 (7.1%)	29 (5.3%)	
Iatrogenic	7 (25.0%)	18 (3.3%)	
Trauma	0 (0.0%)	15 (2.7%)	
From esophagus	2 (7.1%)	4 (0.7%)	
Others	2 (7.1%)	21 (3.8%)	
Lab data			
WBC, mean ± SD (/μL)	13,792.86 ± 7,126.06	14,302.93 ± 6,868.36	0.702
ANC, mean ± SD (/μL)	12,320.34 ± 6,695.18	12,066.21 ± 6,248.99	0.834

*CCI* Charlson Comorbidity Index, *WBC* white blood cell, *ANC* absolute neutrophil count

Both groups were phase II predominant (82.1% in the fungus group vs. 75.0% in the bacteria group, p = 0.390). The location of empyema was mainly at the right side (71.4% in the fungus group vs. 60.5% in the bacteria group), followed by the left side and bilateral. The empyema in the fungus group predominantly had multiple pathogens (78.6%). On the contrary, the empyema in the bacteria group mainly had a single pathogen (72.8%). The causes of empyema varied in concentration between the two groups. Most of the cases in the bacteria group were caused by pneumonia (81.0%), whereas in the fungus group 46.4% of the cases were caused by pneumonia and 25% of the cases were iatrogenic. Most of the iatrogenic fungal empyema was resulted from the surgery of esophageal cancer with esophageal reconstruction. It is believed that this empyema originated from gastric-esophageal anastomosis leakage which contained Candida species from gastrointestinal tract. In terms of white blood cell (WBC) count and absolute neutrophil count (ANC), both groups were similar.

Table 2 shows the clinicopathological characteristics after 1:3 propensity score matching. There were 28 patients with fungal empyema and 84 patients with bacterial empyema. The only factors remained significantly different after matching was the cause of empyema. The distribution of fungus isolates in 28 patients from culture is shown in the Additional file 1: Fig. S1. Twenty-five patients (89%) had *Candida spp.*, 2 patients had *Aspergillus spp.*, and the other patient had *Cryptococcus spp.* The Candida empyema was prescribed with fluconazole alone for average duration of 21 days after diagnosis. The Aspergillus empyema was treated with voriconazole for 8 weeks while the Cryptococcus empyema with oral fluconazole for 6 months.

**Table 2:** Patient characteristics after 1:3 propensity score matching

	Fungal empyema	Bacterial empyema	*P*
Number of patients	28	84	
Age (years) mean ± SD	65.50 ± 11.91	62.25 ± 15.15	0.304
Gender			0.171
Male	26 (92.9%)	69 (82.1%)	
Female	2 (7.1%)	15 (17.9%)	
Smoking			0.663
Never	15 (53.6%)	41 (48.8%)	
Ever	13 (46.4%)	43 (51.2%)	
CCI score			0.876
0	3 (10.7%)	9 (10.7%)	
1 ~ 2	8 (28.6%)	20 (23.8%)	
> = 3	17 (60.7%)	55 (65.5%)	
Phase			0.180
II	23 (82.1%)	58 (69.0%)	
III	5 (17.9%)	26 (31.0%)	
Location			0.497
Right	20 (71.4%)	64 (76.2%)	
Left	6 (21.5%)	18 (21.4%)	
Bilateral	2 (7.1%)	2 (2.4%)	
Pathogen			0.615
Single	6 (21.4%)	19 (22.6%)	
Multiple	22 (78.6%)	65 (77.4%)	
Cause			< 0.001
Pneumonia	13 (46.5%)	73 (86.9%)	
From abdomen	1 (3.6%)	4 (4.8%)	
From neck/mediastinum	1 (3.6%)	0 (0.0%)	
Cancer related	2 (7.1%)	4 (4.8%)	
Iatrogenic	7 (25.0%)	1 (1.2%)	
Trauma	0 (0.0%)	0 (0.0%)	
From esophagus	2 (7.1%)	0 (0.0%)	
Others	2 (7.1%)	2 (2.4%)	
Lab data			
WBC, mean ± SD (/μL)	13,792.86 ± 7,126.06	14,018.93 ± 6,512.62	0.877
ANC, mean ± SD (/μL)	12,320.34 ± 6,695.18	11,904.51 ± 6,154.83	0.763

*CCI* Charlson Comorbidity Index, *WBC* white blood cell, *ANC* absolute neutrophil count

Surgical outcomes after 1:3 propensity score matching are presented in Table 3. The fungal empyema group showed a significantly longer ICU stay than the bacterial empyema group (16 days vs. 3 days, p = 0.002). The median ventilator usage duration was 20.5 days in the fungus group and 3 days in the bacteria group (p = 0.002). The hospital stay duration was also longer in the fungal empyema group (40 days vs. 17.5 days, p < 0.001). The 30-day mortality rate was 21.4% in the fungus group and 5.9% in the bacteria group (p < 0.001).

**Table 3:** Outcomes of surgery between fungal empyema and bacterial empyema after 1:3 propensity matching

	Fungal empyema (n = 28)	Bacterial empyema (n = 84)	*P*
ICU duration, median (IQR) (days)	16.00 (0.50–24.75)	3.50 (0–11.00)	0.002
Ventilator duration, median (IQR) (days)	20.50 (0.50–31.75)	3.00 (0–11.00)	0.002
Hospital duration, median (IQR) (days)	40.00 (22.50–53.00)	17.50 (12.00–35.25)	< 0.001
30-day mortality	6 (21.4%)	5 (5.9%)	< 0.001

*ICU* intensive care unit, *IQR* interquartile range

Both the univariable and multivariable linear regression models for 1-year survival before matching were analyzed, and the results are given in Table 4. Fungal pathogens, advanced age, higher CCI score, and phase III empyema were found to be statistically associated with a worse survival in univariable analysis. In multivariable analysis, fungal pathogens, advanced age, and higher CCI score still remained significant factors.

**Table 4:** Univariate and multivariate analysis for 5-year survival (before matching n = 575)

	Univariate		Multivariate	
	HR (95% CI)	*P*	HR (95% CI)	*P*
Pathogen				
Bacteria (ref)	1		1	
Fungus	2.41 (1.44–4.02)	0.001	1.91 (1.10–3.33)	0.022
Age (per year)	1.03 (1.02–1.04)	< .001	1.02 (1.01–1.03)	< .001
Gender				
Male (ref)	1		1	
Female	0.94 (0.67–1.33)	0.737	0.96 (0.67–1.39)	0.830
Smoking				
Never (ref)	1		1	
Ever	0.97 (0.71–1.33)	0.872	0.98 (0.70–1.38)	0.922
Charlson score				
0 (ref)	1		1	
1 ~ 2	2.56 (1.24–5.29)	0.011	2.27 (1.09–4.71)	0.028
> = 3	7.21 (3.67–14.14)	< .001	5.51(2.78–10.92)	< .001
Phase				
II (ref)	1		1	
III	1.40 (1.03–1.91)	0.032	1.26 (0.92–1.73)	0.155
Location				
Right (ref)	1		1	
Left	0.74 (0.55–1.00)	0.050	0.84 (0.62–1.15)	0.286
Bilateral	1.33 (0.42–4.17)	0.628	1.03 (0.31–3.42)	0.963
Pathogen				
Single	1		1	
Multiple	0.83 (0.59–1.16)	0.268	0.91 (0.64–1.29)	0.590

*HR* hazard ratio, *CI* confidence interval

**Fig. 1:**
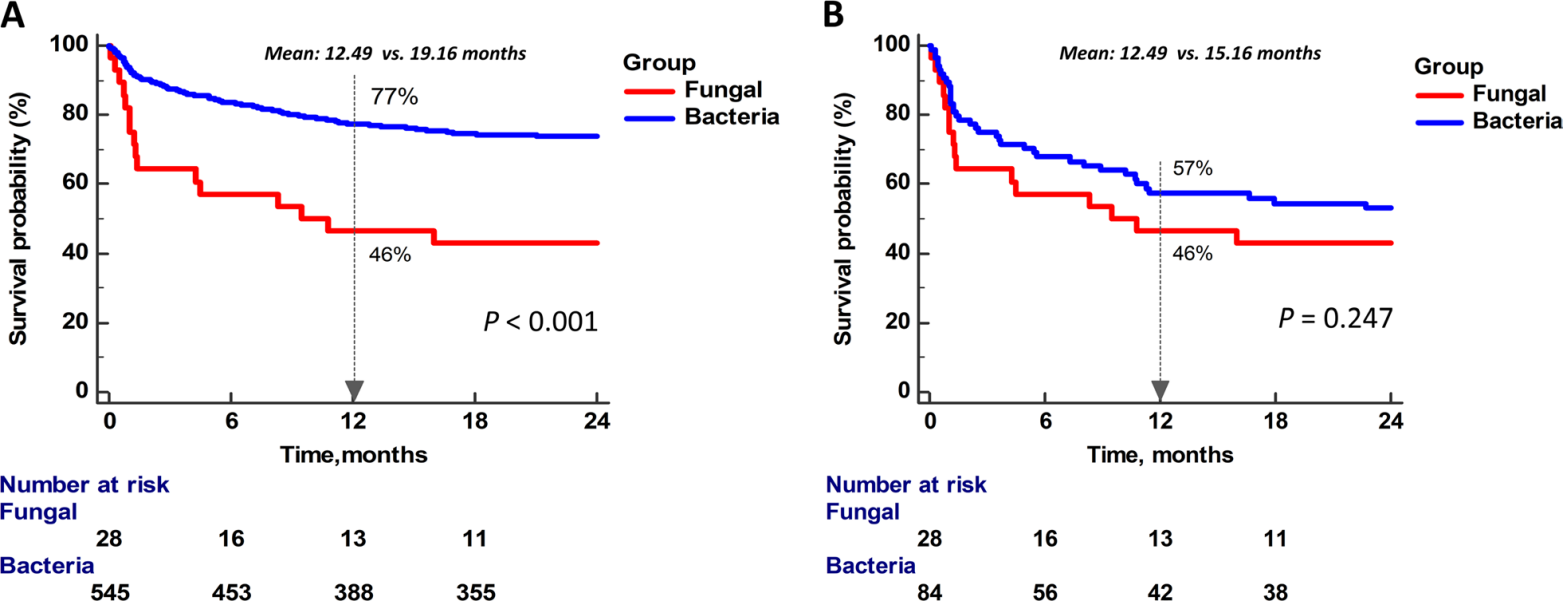
Kaplan–Meier survival curves of fungal empyema and bacterial empyema (**a**) before matching and (**b**) after 1:3 propensity score matching

Figure 1 shows the Kaplan–Meier survival curves for the fungal empyema and bacterial empyema patients. The fungus group revealed significantly poorer 1-year survival than the bacteria group before matching (p < 0.001, Fig. 1a). The mean survival time was 12.49 months in fungus group and 19.16 months in bacteria group. There was no significant difference after matching (p = 0.247, Fig. 1b).

## Discussion

Our study is the first study to compare the surgical outcomes between fungal and bacterial empyema. For all the 1197 patients received surgery, only 28 patients (2.3%) showed fungal empyema while 547 patients (47.5%) revealed bacterial empyema. This data is compatible to a previous study [7]. Our previous study showed that combining a pleural peels tissue culture with a pleural fluid culture during operation could elevate the positive culture rate from 46.0 to 62.7% [10]. However, this method seemed to provide limited effect for fungal empyema. The low positive culture rate is still a problem for the treatment of latent fungal empyema.

In our data, the 1-year survival rate was significantly worse in the fungal empyema group than in the bacterial empyema group before matching. The existence of fungus, advanced age, and a higher CCI score were independent predictors for poor prognosis. The poor prognosis of fungal empyema may be due to etiology, health conditions and lack of proper treatment. First, the common etiologies of fungal empyema differ from those of bacterial empyema. The three most common etiologies of fungal empyema have been reported to be thoracic procedures or trauma, followed by an intra-abdominal source and esophageal rupture [7]. The esophagus is often colonized with *Candida*. If it spreads to pleural space after rupture, there is an increased risk of death [11]. Therefore, isolation of *Candida* species can be a red flag for suspecting gastrointestinal tract perforation as a cause of empyema [12].

Second, health conditions seemed worse in the fungal empyema group than in the bacterial empyema group before propensity score matching. Mild advanced age, a larger proportion of ever smokers, and higher CCI scores were noted in the fungal empyema group, although not all the differences were statistically significant. This can explain why the 1-year survival rate was significantly different before matching but not significantly different after matching. The presence of fungus in a culture indicated a patient’s poor condition and predicted a worse outcome.

Third, the lack of proper treatment for fungal empyema leads to poor outcomes. A majority of the fungal empyema cases had pathogens that were *Candida spp.* Fluconazole has been used most often among patients in the treatment of candidemia. Echinocandins is another treatment choice. However, some data supported the effectiveness of these drugs against Candida empyema and showed a low percentage of penetration into the pleural space [13, 14]. For the treatment of *Aspergillus* empyema, voriconazole and micafungin were reported to provide good pleural penetration and successful treatment [15, 16]. However, most other antifungal drugs have poor pleural concentration [17]. A combined treatment of prompt surgical intervention for drainage promptly and the use of antifungal agents was the primary treatment choice for fungal empyema. Although most of the pathogens for fungal empyema were *Candida* species, one study reported more than fifty percent of cases were complicated by bacterial co-infections [4]. Our data reported a co-infection rate as high as 78.6%. Therefore, each case should be studied individually, and the role of antifungal agents must be evaluated to optimize treatment [5].

We noticed a male predominance, especially regarding fungal empyema. In the fungal empyema group, 92.9% of the patients were male, compared to 77.9% in the bacterial empyema group. A previous study showed similar results and suggested this may be caused by the male-dominant diseases of esophageal cancer and gastric cancer [6]. Iatrogenic gastrointestinal tract perforation or leakage is a notable source of fungal empyema.

This study is the first study to report the outcomes of surgeries for fungal empyema. We demonstrated that fungal empyema indicated poor surgical outcomes and prognoses. There are some limitations of our study. First, this retrospective study may have selection bias, which could affect the data analysis. Second, the isolation of fungal empyema is still scanty. In this study there were only 28 patients with fungal empyema who had positive cultures after surgery. Third, there was limited data about perioperative shock status. It was reported that it may also be an individual risk factor for mortality [6]. The status of malnutrition and immune condition are also lack of data in this retrospective study. They are also important variable and may affect the results.

## Conclusions

Fungal empyema has a rare disease with a high mortality rate. Fungal empyema patients with surgery have much more 30-day mortality and poorer 1-year survival before matching than bacterial empyema patients. Advanced age and a higher CCI score are independent predictors for poor prognosis. A majority of the pathogens are of the *Candida* species. *Candida* empyema may indicate gastrointestinal tract perforation or leakage.

## Supplementary Information

**Additional file 1: Table S1.** The duration of antibiotics duration before surgery and detailed in CCI score. **Fig. S1.** Distribution of fungus isolates from culture

## Data Availability

The datasets used and/or analysed during the current study are available from the corresponding author on reasonable request.

## Abbreviations

CCI: Charlson Comorbidity Index
OS: Overall survival
ICU: Intensive care unit
WBC: White blood cell
ANC: Absolute neutrophil count
