# Supplementary material for: The outcomes of thoracoscopic decortication between fungal empyema and bacterial empyema
Source: BMC Infect Dis. 2023 Jan 6;23:8. doi: 10.1186/s12879-022-07978-z (PMC9817236; doi:10.1186/s12879-022-07978-z)

Additional file

Table S1: The duration of antibiotics duration before surgery and detailed in CCI score

|  | Fungal empyema (n=28) | Bacterial empyema (n=547) | *P* |
| --- | --- | --- | --- |
| Antibiotics duration before surgery, median (IQR) (days) | 8.00(3.25-15.00) | 3.00(1.00-6.00) | <0.001 |
| Cancer | 15(53.6%) | 171(31.3%) | 0.014 |
| Diabetes | 10(35.7%) | 201(36.7%) | 0.912 |
| Renal insufficiency/failure | 2(7.1%) | 46(8.4%) | >0.999 |
| Hepatic failure | 2(7.1%) | 3(0.5%) | 0.021 |

CCI: Charlson Comorbidity Index

Figure S1: Distribution of fungus isolates from culture


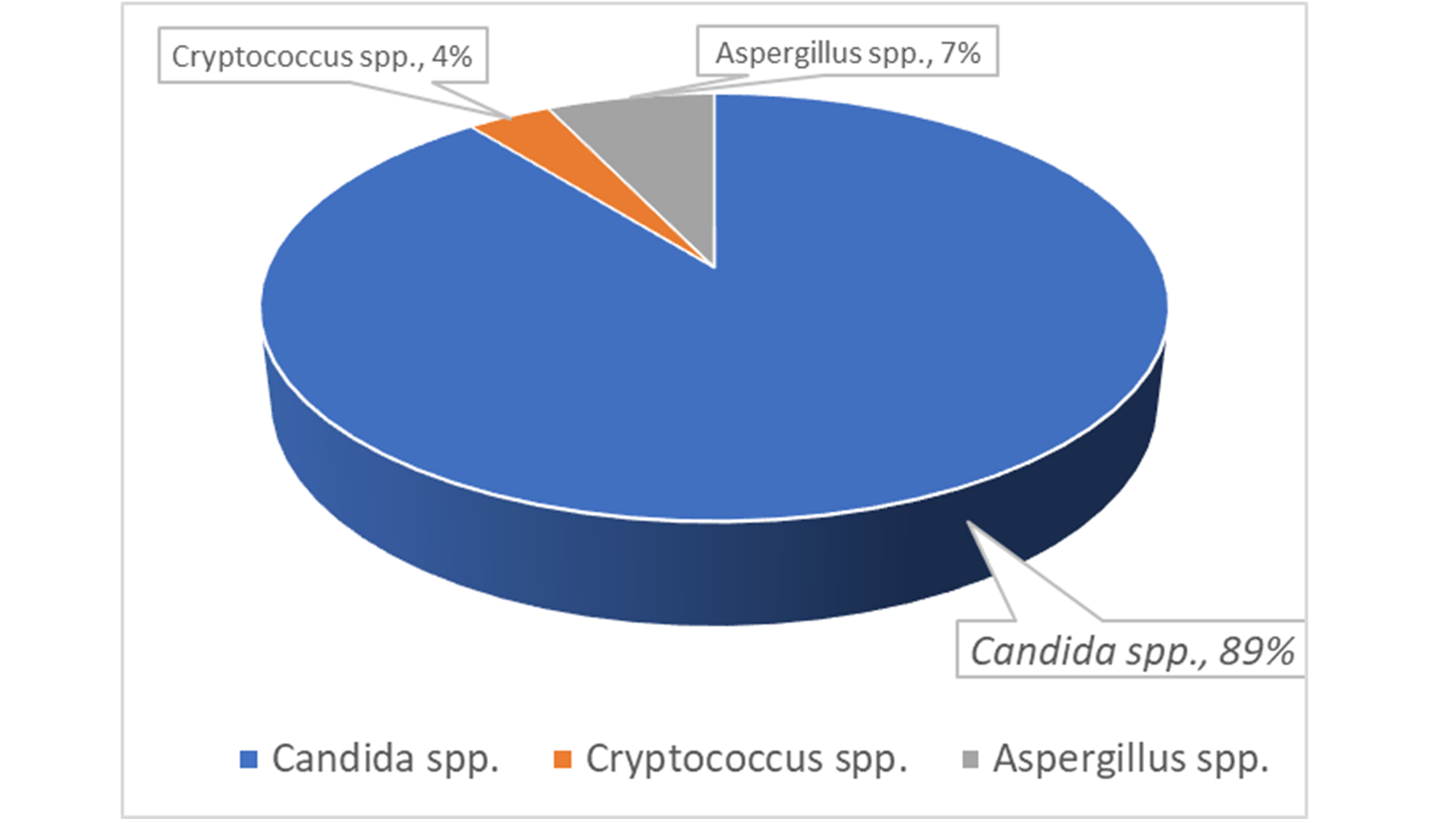

Supplement: Supplementary file 1 — Additional file 1: Table S1. The duration of antibiotics duration before surgery and detailed in CCI score. Fig. S1. Distribution of fungus isolates from culture [file 12879_2022_7978_MOESM1_ESM.doc]
